# Optical coherent dot-product chip for sophisticated deep learning regression

**DOI:** 10.1038/s41377-021-00666-8

**Published:** 2021-11-01

**Authors:** Shaofu Xu, Jing Wang, Haowen Shu, Zhike Zhang, Sicheng Yi, Bowen Bai, Xingjun Wang, Jianguo Liu, Weiwen Zou

**Affiliations:** 1 State Key Laboratory of Advanced Optical Communication Systems and Networks, Intelligent Microwave Lightwave Integration Innovation Center (imLic), Department of Electronic Engineering, Shanghai Jiao Tong University, 800 Dongchuan Road, Shanghai, 200240 China; 2 State Key Laboratory of Advanced Optical Communications System and Networks, Department of Electronics, School of Electronics Engineering and Computer Science, Peking University, Beijing, 100871 China; 3 Institution of Semiconductors, Chinese Academy of Sciences, Beijing, 100083 China

**Keywords:** Optoelectronic devices and components, Photonic devices

## Abstract

Optical implementations of neural networks (ONNs) herald the next-generation high-speed and energy-efficient deep learning computing by harnessing the technical advantages of large bandwidth and high parallelism of optics. However, due to the problems of the incomplete numerical domain, limited hardware scale, or inadequate numerical accuracy, the majority of existing ONNs were studied for basic classification tasks. Given that regression is a fundamental form of deep learning and accounts for a large part of current artificial intelligence applications, it is necessary to master deep learning regression for further development and deployment of ONNs. Here, we demonstrate a silicon-based optical coherent dot-product chip (OCDC) capable of completing deep learning regression tasks. The OCDC adopts optical fields to carry out operations in the complete real-value domain instead of in only the positive domain. Via reusing, a single chip conducts matrix multiplications and convolutions in neural networks of any complexity. Also, hardware deviations are compensated via in-situ backpropagation control provided the simplicity of chip architecture. Therefore, the OCDC meets the requirements for sophisticated regression tasks and we successfully demonstrate a representative neural network, the AUTOMAP (a cutting-edge neural network model for image reconstruction). The quality of reconstructed images by the OCDC and a 32-bit digital computer is comparable. To the best of our knowledge, there is no precedent of performing such state-of-the-art regression tasks on ONN chips. It is anticipated that the OCDC can promote the novel accomplishment of ONNs in modern AI applications including autonomous driving, natural language processing, and scientific study.

## Abstract (web summary)

An optical coherent chip completes state-of-the-art image reconstruction tasks with 32-bit computer comparable image quality, showing potential in conquering sophisticated deep learning regression tasks.

## Graphical abstract

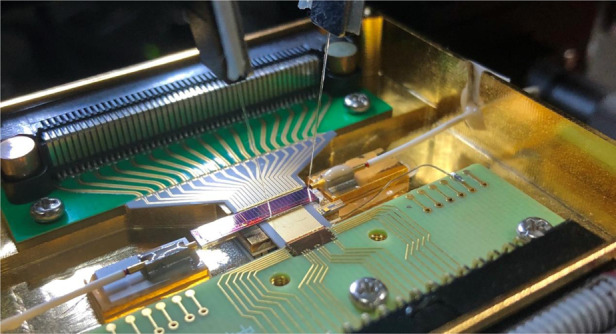

## Introduction

Because of the flourishment of artificial intelligence (AI), we witness the revolution of technical foundations of emerging applications, such as autonomous driving, natural language processing, and medical diagnosis^1–3^. In addition, profound insights are offered to scientific studies across disciplines such as chemistry^4^, physics^5,6^, and biomedicine^7^. One of the major driving forces of AI is the blooming of artificial neural networks (ANNs), which are mathematically composed of thousands of nodes and millions of interconnections layer by layer. A high-dimension representation space is thus supported by the large-scale neural network. Such large representation space of ANNs enables high-volume automatic feature extraction from original data, so that intricate transformations of chemical, physical, and biological systems can be precisely fitted and predicted. However, large representation space demands massive computational costs. Currently, Moore’s law of integrated circuits is slowing down^8^ while the scale expansion of ANNs is speeding up^9^. The compute capability of conventional digital computers is falling behind. To solve the problem, optical implementations of neural networks (ONNs) have been recently proposed and demonstrated to realize high-speed and energy-efficient AI hardware^10–14^. Linear propagation of light equivalently carries out the linear computing of ANNs^15–19^; ultra-wide optical transparent spectrum and high-speed modulators/detectors enable a fast clock rate (tens of GHz)^20,21^; and non-volatile photonic memory makes the computing “zero-consuming”^22^.

However, a substantial improvement of ONNs needs to be achieved for accomplishing state-of-the-art AI applications. For now, ONNs are mostly demonstrated with classification tasks on elementary datasets such as MNIST handwritten digit recognition^23^ because of their simplicity for primary validation. As an important form of deep learning, regression tasks, such as image reconstruction^24^, machine gaming^25^, and nanostructure design^26,27^, remain uninvestigated. Distinct from classification, regression demands the neural network to output continuous values instead of discrete categories. Carrying out computations in the complete real-value domain with high numerical accuracy is the basic requirement for regression, which is still challenging for the existing ONN chips. Firstly, for non-coherent ONN architectures^16,20–22^, input values are represented by non-negative optical intensities, causing incompleteness of the numerical domain. In contrast, coherent ONN architectures^15,28,29^ adopt optical fields to represent real-valued inputs and homodyne detection to yield real-valued outputs, showing the capability of computing in the complex-valued domain. Nonetheless, the size of existing ONN chips is much smaller than that of regression neural networks, and the complexity of chip calibration for coherent ONNs increases the difficulty of reaching high numerical accuracy. Therefore, high-quality deep learning regression still remains challenging in the ONN field.

Here, we propose and experimentally demonstrate a silicon-based optical coherent dot-product chip (OCDC) to implement sophisticated regression tasks. Values are modulated into the amplitudes of optical fields and the output field is read out via optical interference. In this sense, it is feasible to operate the OCDC in the complete real-value domain. The chip is reconfigured to conduct linear operations including matrix multiplications and convolutions, and it is reused to carry out arbitrarily sophisticated neural networks. As the OCDC is an analog computing device, parameters represented by nonideal hardware often deviate from desired ones. The simple architecture of the OCDC enables compensation for such deviations by in-situ backpropagation control (BPC), thus obviously enhancing numerical accuracy. With these properties, the chip meets the basic prerequisites for deep learning regression tasks. We benchmark the OCDC with a neural network, AUTOMAP^30^, which achieves state-of-the-art performance in image reconstruction. The experimental result verifies that the OCDC can effectively compute both fully connected (FC) layers and convolutional layers, covering all linear operations required by most neural networks. The performance of OCDC in the AUTOMAP image reconstruction task is comparable with that of a 32-bit digital computer. To the best of our knowledge, it is the first time demonstrating sophisticated deep learning regression tasks with on-chip optical computing hardware. The insights provided by this work are inspiring for further investigations on practically applicable ONNs.

**Fig. 1:**
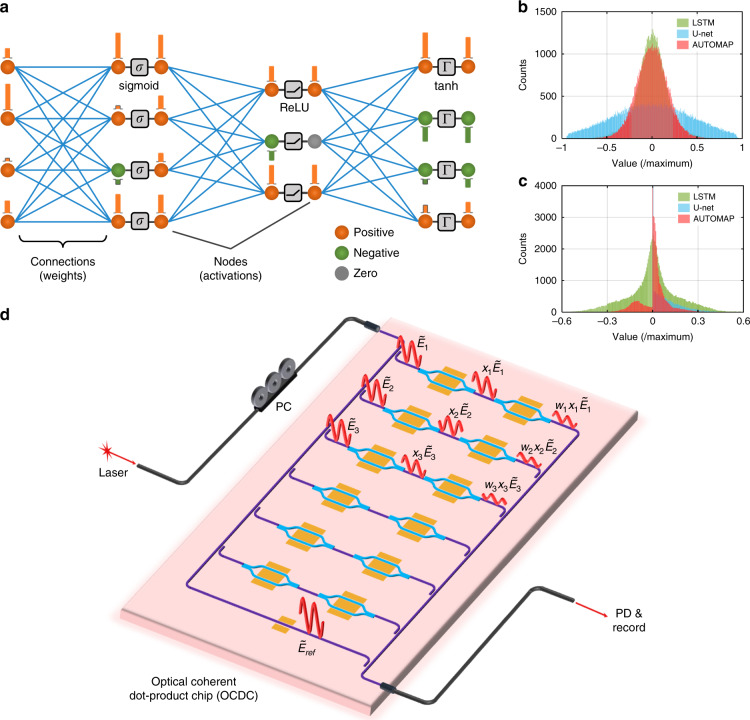
Schematic of the optical coherent dot-product chip (OCDC). **a** Simplified model of a neural network for regression. A neural network is typically composed of nodes, connections, and activation functions. Three typical functions (sigmoid, rectified linear unit (ReLU), and hyperbolic tangent (tanh)) are depicted in the figure. Positive, negative, and zeros nodes are depicted with different colors. For fully connected networks, a node represents a single value and a connection is a weight. For convolutional networks, a node is a feature map and a connection is a convolutional kernel. **b** Histograms of weights in LSTM^31^, U-net^32^, and AUTOMAP^30^, respectively. They approximately obey normal distribution with a mean value of zero. **c** Histograms of activated values in LSTM, U-net, and AUTOMAP, respectively. The distribution depends on the activation functions being used. **d** The conceptual schematic of the OCDC. It contains several parallel branches for dot product and one extra branch for coherent detection. The optical field in each branch is symbolized with red curves. The push-pull configured modulator imposes amplitude-only modulation to the optical field without introducing phase shift. Hence, the phase of each branch is stable

In Fig. 1a, a simplified model of an ANN for regression tasks is depicted. The network is composed of nodes and connections layer by layer. A node represents a single value in FC layers or a feature map in convolutional (conv.) layers. Connections represent the weight matrix or convolutional kernels for FC layer or conv. layer correspondingly. Each layer contains a linear part, i.e., matrix–vector multiplication (MVM) and convolution, and a nonlinear activation function to obtain the activated values. From left to right, the input nodes are calculated layer by layer to yield the final output nodes. For regression tasks, weights in the network are trained to minimize the distance between the final outputs and the ground truth (data deemed as reference). In general cases of regression networks, the numerical basis for both activated values and weights is the real-value domain. In Fig. 1b, c, we illustrate the histograms of weights and activated values of three well-known regression ANNs, long short-term memory (LSTM)^31^, U-net^32^, and AUTOMAP^30^. The overall distribution of trained weights in these ANNs always obeys normal distributions and the mean values are near zero. Negative weights are as many as positive ones. The activated values perform diversely. In the U-net with the ReLU activation function, activated values are non-negative. For this kind of neural network, only positive input values are need and non-coherent ONN architecture performs similarly with coherent architectures. However, the LSTM network and the AUTOMAP, which are based on hybrid sigmoid, ReLU, and tanh functions, contain both positive and negative activated values. Therefore, the capability of representing real activated values and weights is a basic requirement for the optical implementation of regression networks. Also, we note that the dot product is a building block of MVMs or convolutions. Building hardware for dot products enables equivalent calculation of the linear part of ANNs. Following these guidelines, we design the OCDC as shown in Fig. 1d. To keep the signs of activated values and weights, optical amplitude modulation is adopted and the output amplitude is detected via coherent interference. The light from a coherent laser source is split into M + 1 branches, with M branches performing the dot product and the last one being the local reference for coherent detection. Optical power is evenly distributed in these M branches. Inside each branch, two modulators under push-pull configuration are deployed serially to impose optical amplitude modulation without introducing extra phase shift. As the first one represents the value of *x*_*i*_ and the next one represents *w*_*i*_, the output is the multiplication of these two values^18^. When all branches match in phase, the optical fields interfere constructively in the optical combiner to complete summation. Before photodetection, the local reference is combined with the summed optical field, introducing an amplitude bias to avoid the elimination of negative amplitude at photodetection. The process of the OCDC can be formulated as the following equation.

1 $$\begin{array}{ll}I_{\rm{photo}} \propto {\mathbf{Re}} \left\{ {\left( {\tilde E_{\rm{ref}} + \mathop {\sum}\limits_i {w_ix_i\tilde E_i} } \right)\left( {\tilde E_{\rm{ref}} + \mathop {\sum}\limits_i {w_ix_i\tilde E_i} } \right)^ \ast } \right\}\\ \qquad\quad= \left\| {\tilde E_{\rm{ref}} + \mathop {\sum}\limits_i {w_ix_i\tilde E_i} } \right\|^2\end{array}$$

When the amplitude of local reference $\tilde E_{\rm{ref}}$ is larger than the weighted sum, the sign of dot product is maintained after photodetection.

## Results

**Fig. 2:**
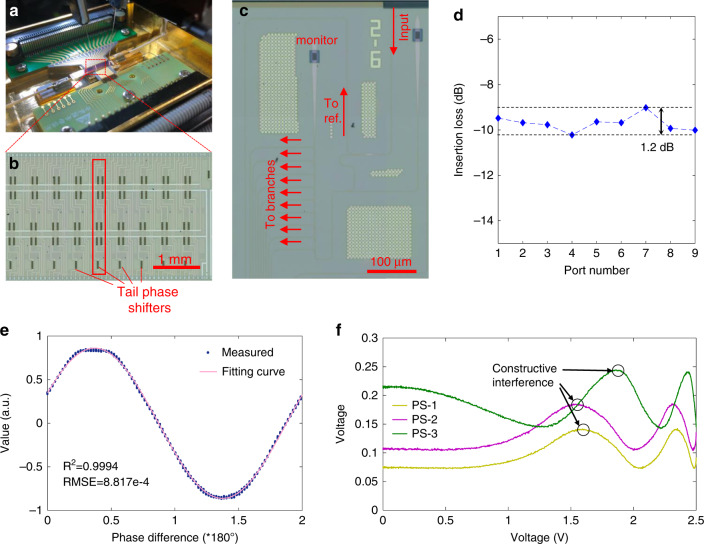
Chip characterization results. **a** The packaged OCDC with periphery circuits. **b** The top view of the OCDC. Modulating branches are located vertically as the red block shows. Every modulator contains four phase shifters, two of which are used to conduct push–pull modulation and the remaining two are used to control the bias voltage. Tail phase shifters are appended to compensate for the phase difference among branches. **c** The structure of light input and splitter. **d** Characterization of evenness of optical power splitting. **e** Modulator characterization with push–pull driven. The experimental result is fitted with a curve formulated by *a* * sin(*b* * *x* + *c*) + *d*. The information on fitting is also given in the plot. **f** The effect of constructive interference of three branches. Three curves are obtained one by one. Firstly, only the first branch is modulated to the highest transparency when the other two branches are closed. By changing the voltage on its tail phase shifter (PS-1), the yellow curve is shown. It denotes the interference result of branch 1 and reference branch. Secondly, keep the voltage on the PS-1 at the constructive interference point (black circled); modulate the second branch to its highest transparency; change the second tail phase shifter (PS-2). The purple curve is recorded. By operating the same process for the third branch, we obtain the green curve

The OCDC is fabricated with a silicon-on-insulator (SOI) process. Fig. 2a shows the packaged OCDC and its periphery circuits. Common metal wires on the bottom provide voltages for the chip. Transmission lines on the top are used to transfer high-speed signals. The layout of the chip is shown in Fig. 2b. Optical splitters, push-pull modulators, combiners, couplers are systematically integrated on the chip. Two optical splitters (shown in Fig. 2c) are used to divide optical power into the nine modulating branches and the reference. A multi-mode interferometer split half of the optical power to the reference. The left power is split into nine branches by cascaded directional couplers (DCs). The coupling length of each DC is designed so that the optical power is divided evenly. Figure 2d provides the measured splitting ratio of the cascaded DCs, showing an evenness lower than 1.2 dB. At each modulating branch, we fabricate a tail phase shifter to compensate for the phase difference between branches and the reference. More details of chip fabrication and characterization are provided in the Supplementary Section. In the OCDC, stable push–pull modulation is important to keep the constructive interference for photodetection. Figure 2e shows an example result of push-pull modulation (see “Methods”). This result is yielded by complementarily changing voltages on the upper and lower arms of a single modulator with other modulators staying static. The amplitude of the output optical field varies along a cosine curve when the applied voltages change. The *R*^2^ of the fitting is 0.9994, indicating that the push–pull modulation is accomplished with high stability. Then the constructive interference of multiple branches is inspected in Fig. 2f. It is shown that, with proper configuration of the tail phase shifter, the output optical fields of different branches can match in-phase, performing a jointly constructive interference. Implied by the results, the OCDC is capable of amplitude modulation and coherent detection, laying the basis for calculations of the real-valued dot products.

**Fig. 3:**
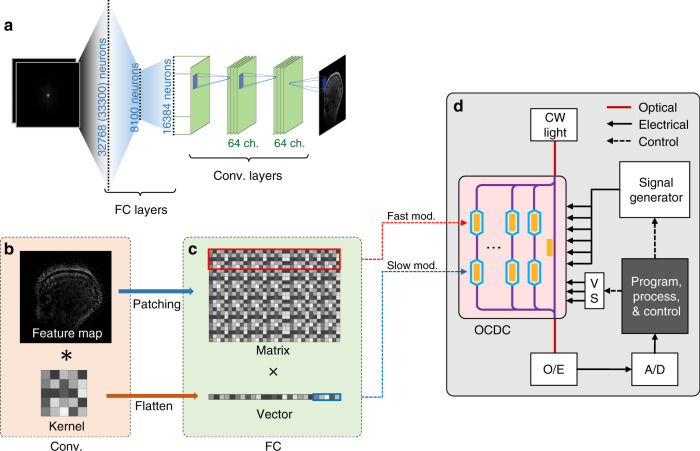
Experimental setup of OCDC validation. **a** The network structure of the AUTOMAP. The input images are flattened into vectors and process with two fully connected layers. The numbers of neurons are 32,768 (or 33,300), 8100, and 16,384, respectively. Then, the output neurons are converted back to feature maps sized 128 × 128. Two convolutional layers and a de-convolutional layer process this feature map to the output image. The number of channels in convolutional layers is 64. **b** The process of convolutions. After patching and flattening, convolutions can be converted to MVMs. **c** The process of MVM. The matrix and the vector are loaded onto the fast mod and slow mod., respectively. The red and blue blocks show the decomposition of the input vector and matrix. **d** Experimental setup of OCDC validation. CW light continuous-wave light source, O/E optoelectronic detection, A/D analog-to-digital converter

We implement AUTOMAP as a representative example of deep learning regression to validate the OCDC. Figure 3a shows the structure of the AUTOMAP containing two FC layers, two convolutional layers, and a de-convolutional layer. Details of the neural network can be found at the ref. ^30^. and “Methods”. Given that the linear part of FC layers and convolutional layers can be decomposed to dot products; we can realize these layers by reusing the OCDC temporally. Figure 3b–d shows the method of conducting MVMs and convolutions via temporal multiplexing. The decomposition of MVMs is straightforward since they are naturally calculated via vector-vector dot product. The input vector is loaded onto the second row of modulators marked “slow mod.”, and a vector from the matrix is modulated onto the “fast mod.” modulators. By temporally changing the vectors loaded on the “fast mod.” modulators, the result of MVM is eventually calculated. When the size of vectors is too large to be loaded onto these modulators in one time, the vector and the matrix can be divided into small parts as depicted in Fig. 3c. For convolutions, the process of patching^33,34^ can rearrange pixels of the feature map into a matrix. The kernel is flattened as a vector. In this way, MVMs and convolutions can be similarly conducted by the OCDC. Figure 3d is the experimental setup (detailed in “Methods”) for temporally multiplexing the OCDC. A signal generator (max. bandwidth is 20 MHz) is used to provide signals for amplitude modulation and a voltage source (VS) supplies the bias voltages. A computer (the gray block) is adopted to carry out programs. It controls the signal generator and the VS to work as a whole. It also records and processes the output data from the OCDC.

**Fig. 4:**
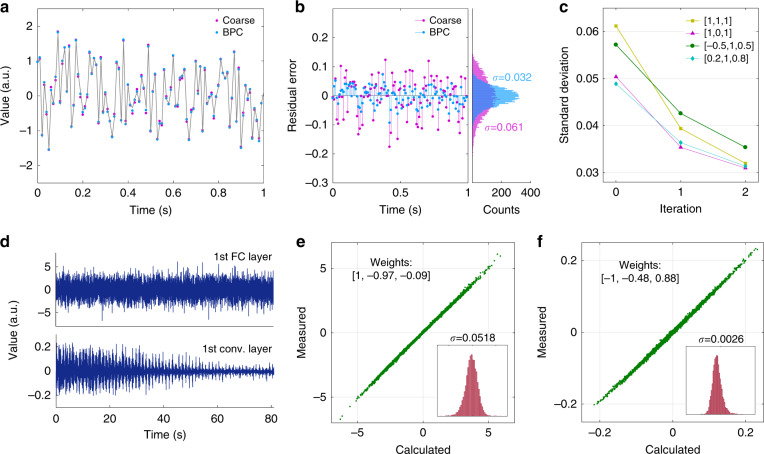
Experimental results of OCDC validation. **a** Samples before and after the BPC. The “coarse” samples are yielded with the coarsely calibrated hardware. The weights adopted is [1, 1, 1]. The gray curve shows the correct result calculated by a computer. **b** Residual error of the samples before and after the BPC process. Histograms and standard deviations (*σ*) are also attached on the right side. **c** BPC process with different weight combinations. **d** Output waveforms of the OCDC conducting the first FC layer and the first convolutional layer, respectively. **e**, **f** Scatter plots of the measured samples vs. calculated expectation (FC layer and conv. layer, respectively). Corresponding weights are marked on the top-left. A histogram of residual error is attached on the bottom-right

As analog computing hardware, the OCDC suffers from the imperfectness of fabricated devices. The actual values represented by the analog devices often deviate from the desired ones. Such deviations come from multiple sources including uneven splitter, combiner, modulation efficiency, and phase drift. Distinct from classification tasks, deep learning regression asks for higher numerical accuracy since it directly corresponds to the quality of regression (see Fig. S9 for more information). Therefore, we adopt an in-situ BPC method to minimize such deviations (see “Methods”). Figure 4a, b shows the effectiveness of the BPC with an example of random inputs. The weights adopted is [1, 1, 1]. Although the modulators are coarsely calibrated before conducting dot products, the numerical accuracy is insufficient for high-quality deep learning regression. After the BPC, the accuracy of the analog computing is improved with residual error dropping from 0.061 to 0.032. With more weight combinations (see Fig. 4c and Fig. S10), we validate that the BPC can increase the numerical accuracy of the coarsely calibrated hardware within two iterations.

Based on the BPC method, we experimentally implement the first FC layer and the first convolutional layer of AUTOMAP using the OCDC. Due to the limited hardware scale, the parameters are trained on a computer and the OCDC is used for inference (see the “Methods” for details of network training). For the FC layer, the sizes of the input vector (1 × 32768) and the weight matrix (32768 × 8100) are massive. We decompose the vector and the matrix to the size of 1 × 3 and 3 × 8100, respectively, according to the size of the OCDC. For the convolutional layer, the input feature map (128 × 128) is firstly patched to a matrix (25 × 16384) and the kernel (5 × 5) is flattened. Similarly, we decompose the massive matrix into small parts for the feasibility of the OCDC. The OCDC carries out the linear parts and the nonlinear activation functions are implemented in the computer. Further details of the experiment are provided in “Methods”. An example result is shown in Fig. 4d. Each temporal waveform contains 8100 samples since the OCDC is temporally multiplexed by 8100 times. Limited by the bandwidth of the thermo-optic modulator and the signal generator, the modulation rate is 100 Hz. A high-speed OCDC is further discussed in Supplementary Section. In Fig. 4e, f, the computing accuracy of the FC layer and the convolutional layer are inspected, respectively. We observe that the measured samples tightly concentrate at the diagonal line where indicates correct results. The normalized standard deviation of the residual error is 0.0518/7.0 = 0.0074 and 0.0026/0.25 = 0.0104, respectively. Such results are competitive among coherent ONN architectures, around six times less than that reported in ref. ^15^.

**Fig. 5:**
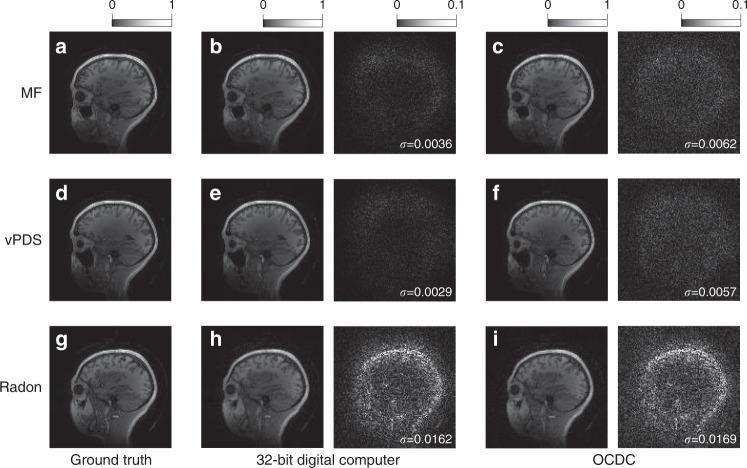
Reconstructed images of the AUTOMAP by a 32-bit computer and the OCDC. **a**, **d**, **g** The ground-truth images with MF, vPDS, and Radon processes, respectively. **b**, **e**, **h** The reconstructed images by the 32-bit computer. Values of the images are normalized to 1. Scale bars are attached on top. The residual error maps are attached to the right. Values of the error are amplified by 10 times for better visibility. Standard deviations (*σ*) are shown on the error maps. **c**, **f**, **i** Reconstructed images of the OCDC, with the same normalization

Then, the task of image reconstruction is demonstrated with the accuracy achieved by the OCDC (details are provided in “Methods”). Example results are shown in Fig. 5 and Figs. S12–14. Since the AUTOMAP is a unified neural network that can reconstruct images from various input formats^30^, we demonstrate three typical reconstruction processes for magnetic resonance imaging (MRI): misaligned Fourier (MF) space^35^, variable Poisson disk sampled (vPDS) Fourier space^36^, and Radon projection^37^ (see “Method” for further details). Figure 5a–c illustrates a comparison of the results yielded by a standard 32-bit computer and the OCDC. The corresponding process is MF. We observe that the OCDC accomplishes image reconstruction with high quality. The standard deviations of image absolute error are 0.0036 and 0.0062 for the digital computer and the OCDC, respectively. Note that the values of error are amplified by 10 times for better visibility. Such an increase of error of the OCDC is hardly visible from the reconstructed image. Figure 5d–f is the reconstructed images of the vPDS process. The quality of the OCDC reconstructed image is also acceptable. For the Radon reconstruction, the performance of the AUTOMAP conducted by the computer is inferior to the previous two processes. The gap between the 32-bit computer and the OCDC is greatly reduced. From the results, we observe that the OCDC can achieve comparable performance with a 32-bit digital computers on image reconstruction, implying its further applications on other regression tasks.

## Discussion

Researches on ONNs are commonly in pursuit of high-speed and energy-efficient computing. Although the main contribution of this work is originally demonstrating regression tasks, we note that the proposed architecture is the potential to be upgraded toward a high-speed one. Recent breakthroughs of electro-optic integrated modulators, on conventional SOI platforms or thin-film lithium niobate platform^38–40^, pave the way for a high modulation rate. Replacing the thermo-optic modulators used in the proof-of-concept (the “fast mod.” part) can greatly enhance the computing speed. In Supplementary material, an electro-optic version of OCDC is measured and discussed. If the OCDC is designed at high speed, the system noise will increase inevitably. The signal-to-noise ratio (SNR) might become a limiting factor to the performance of the OCDC. Generally speaking, SNR is mostly determined by the insertion loss and photodetection noise^12^. If the insertion loss and the photodetection noise are kept low with current advanced waveguide and photodetection technologies, high SNR is achievable at a high modulation rate over 10 GHz and high-quality image reconstruction is also expectable. The OCDC has the potential to be duplicated spatially for exploiting the optical advantage on parallelism (described detailly in Supplementary Section). The operation speed can thus be multiplied and the energy consumption per operation can be lowered^11–13,19^. Moreover, the phase shifters in the “slow mod.” part can be replaced with non-thermal devices such as nano-optical electromechanics^41^, which are “zero-consuming” in static states. Energy consumption can thus be significantly lowered. In the coming future, it is unlikely the complexity of physical ONN systems should surpass that of practical ANNs. The key to implementing sophisticated ANNs, as demonstrated by this work, is temporal multiplexing with the assistance of electronic devices^10^. Thanks to recent progress in hybrid integration of electronics and photonics^42,43^, it is optimistic to build a monolithic system with the OCDC for high-performance computing and affiliated electronics for instructions and memory.

As the prosperity of modern AI relies heavily on the success of deep learning regression, we demonstrate the OCDC to promote the application of ONNs in regression tasks. In our approach, firstly, values in the complete real domain are represented by optical fields. Output values are detected via optical coherent interference to maintain amplitude information. Secondly, the size of neural networks that are used in regression tasks is far larger than that of currently available ONNs. We reconfigure and reuse the OCDC so that matrix multiplications and convolutions of arbitrary size can be equivalently conducted. Thirdly, the OCDC features precise control through the BPC method to reach a high numerical accuracy (normalized deviation of ~0.01). Therefore, the OCDC meets the prerequisite for sophisticated deep learning regression tasks. A state-of-art image reconstruction neural network, AUTOMAP, is adopted to benchmark the proposed chip. Experimental results validate that the OCDC can accomplish AUTOMAP with the comparable performance of the 32-bit digital computer. Since the basic building blocks of ANNs across different applications are similar, we believe the OCDC can be further applied in more advanced AI fields, including autonomous driving, natural language processing, medical diagnosis, and scientific study.

## Materials and methods

### Experimental setup

A brief schematic of the experimental setup is illustrated in Fig. 3d. A continuous-wave laser (Alnair Lab TLG-220) working at 1550 nm is used as the coherent light source. The output of the OCDC is directly linked with the amplifier photodetector (THORLABS PDA10CS2). The electrical signal is digitized and recorded with an oscilloscope (KEYSIGHT DSO-S 804A). A multi-channel arbitrary waveform generator (AWG) is used as the signal source for the OCDC. The AWG contains 9 NI-PXIe-5413 blades, each of which has two output channels, and an embedded computer (NI-PXIe-8880) to control the system. The signals recorded by the oscilloscope are also transmitted to the computer for further processing. In the experiment, we use three branches (six modulators) to demonstrate the OCDC’s feasibility on dot products. Since each modulator requires 2 electrical signals for push-pull driving, 12 output channels of the AWG are adopted. The trigger of the oscilloscope and the AWG are synchronized for stable sampling. A homemade 45-channel VS is used for supplying the bias voltages of the modulators. The control signal is also provided by the embedded computer. As the proof-of-concept, single-ended photodetection is used in this work, so that the photocurrent is quadratic to the optical amplitude. To achieve a linear mapping between the electrical signal and optical amplitude, homodyne photodetection is preferred (see Fig. S8b). Note that the correct operation of OCDC is based on stable coherent interference, a thermo-resistor and a thermo-electric cooler are packaged in the module and are controlled by a temperature controller (Thorlabs ITC4002QCL). The module is stored and measured with indoor humidity of 30–70%. Long-time exposure to high humidity might shift the position of the fiber port, increasing the insertion loss. Therefore, it is preferred to use airtight seals in module packaging.

### Push–pull modulation

In the OCDC architecture, the final output is yielded by constructive interference. It is important to keep the phases of different branches stable to maintain constructive interference. Ordinary single-driven configuration of modulators not only impose amplitude modulation but also introduce extra phase shift. Therefore, the push–pull configuration is necessary for the OCDC. In the experiment, thermo-optic modulators are adopted, where phase shift from the thermal effect is proportional to applied power (quadratic to voltage). Therefore, the relation between applied voltages and the expected phase difference Δ*φ* is formulated as

2 $$V_{\rm{upper}} = \sqrt {\frac{{P_0 + \frac{{\Delta \varphi }}{{2\pi }} \cdot P_\pi }}{R}}\; V_{\rm{lower}} = \sqrt {\frac{{P_0 - \frac{{\Delta \varphi }}{{2\pi }} \cdot P_\pi }}{R}}$$

where *V*_upper_ and *V*_lower_ are the voltage applied to the upper arm and the lower arm of a modulator, respectively. *P*_0_ is a bias power applied to the upper and lower arms at the same time. This bias power allows negative phase shift to the lower arm, thus realize push–pull configuration. *P*_*π*_ is the required power for a 180° phase shift, which is different for every single thermal phase shifter. *R* is the resistance of the thermal phase shifter, which is measured to be 1.6 kΩ. For simplicity, for all thermal phase shifters, the *P*_0_ is set at 2.28 mW, and an approximate value of *P*_*π*_, 1.81 mW, is used. With a proper bias voltage, the transmission function of the modulator is sin(Δ*φ*). With this transmission function, we encode the pixel values in grayscale to voltages for modulation. We have also built an electro-optic (EO) version of OCDC (see Supplementary Section for more information) with silicon-based modulators. To achieve push–pull modulation on these EO modulators, a common positive bias voltage should be applied to the phase shifters of the upper arm and lower arm before the differential voltage signals are applied.

### The architecture of the AUTOMAP

The AUTOMAP is composed of, from first to last, two FC layers, two convolutional layers, and a de-convolutional layer. The input images are flattened to an input vector of the first FC layer. For MF and vPDS processes, the shape of input images is 128 × 128 × 2. Therefore, the length of the input vector is 32,768. For the Radon process, the size of the input image is 185 × 180 = 33,300. The number of output neurons in the first layer and the second layer is 8100 and 16,384, respectively. The original number of neurons in the first layer is 16,384. In our experiment, this number is modified due to the memory limitation of our training platform (NVidia RTX-2080ti with 10.6 GB graphic memory). The activation function of FC layers is “tanh”. After two FC layers, the resultant vector is transformed back to the form of an image with the shape of 128 × 128 for 2D convolutions. The number of output feature maps of these two convolutional layers is 64, and their size is 128 × 128. The kernel size is 5 × 5, and the activation function is ReLU. The kernel size of the de-convolutional layer is 7 × 7. The de-convolutional layer directly outputs the image result without activation.

### Neural network training

The dataset for image reconstruction is assembled from four subjects of the MGH-USC HCP program^44^. Totally, 3100 sagittal scanned brain MRIs are used for neural network training and validation. The original size of these images is 256 × 256, and they are undersampled to 128 × 128 for the capability of the AUTOMAP. The maximal values of these images are normalized to 1 and the mean values are subtracted.

The AUTOMAP is a generally feasible network that can reconstruct images from various processes with the same network hyperparameters. In this work, we demonstrate three processes: reconstructing images from MF spaces^35^, from undersampled Fourier spaces^36^, and from Radon projections^37^.

- The MF process. In MRI, ghost images often occur when the Fourier spaces of two trajectories are physically misaligned. The AUTOMAP is trained to reconstruct images without ghosts from the MF spaces. For training, MF spaces are generated from the original images. The images are firstly transformed to their Fourier space by fast Fourier transform. Then an extra phase shift is added to the even row of the Fourier space. The real part and the imaginary part of the processed Fourier spaces are used as the inputs of the AUTOMAP and the original images are the ground truths for training.
- The undersampled Fourier process. In this process, the AUTOMAP is trained to reconstruct images from the Fourier spaces which have been sparsely undersampled, i.e., only a few pixels are reserved and others are set to zeros. To generate these undersampled Fourier spaces, we adopt the vPDS method with a sparsity of 0.6 to the Fourier spaces transformed from the original images. Again, during training, the real part and the imaginary part of the undersampled Fourier spaces are used as inputs and the original images are used as the ground truths.
- The Radon process. Radon projection is a conventional method for MRI. Here, the AUTOMAP is trained to perform inverse Radon transform with better quality than the conventional one. The Radon projection is generated directly from the original images using discrete Radon transform (180 projection angles with 185 parallel rays). These Radon projections are used as inputs and the original images are the ground truths for training.

The dataset with 3100 examples is randomly divided into a training set (2700 examples) and a validation set (400 examples). The loss function of training is formulated as

3 $$\begin{array}{ll}L^\Theta = \dfrac{1}{{N^2}}\mathop {\sum}\limits_{i = 1}^N {\mathop {\sum}\limits_{j = 1}^N {\left( {y_{i,j}^\Theta - \hat y_{i,j}} \right)^2} } + \dfrac{\lambda }{{KN^2}}\mathop {\sum}\limits_{s = 1}^K {\mathop {\sum}\limits_{i = 1}^N {\mathop {\sum}\limits_{j = 1}^N {\left| {h_{s,i,j}^\Theta } \right|} } }\end{array}$$

where *y*^*Θ*^ is the output image by the neural network and $\hat y$ is the ground truth image. *h*^*Θ*^ represents the output feature maps of the second convolutional layer. *N* = 128 is the width of the images, and *K* = 64 is the number of feature maps. The penalty factor *λ* is 0.0001. The optimization method is the “Adam” optimizer^45^ with a learning rate of 2e−5. After 850 epochs of training, the learning rate is decayed to 2e−6 for better convergence. For different reconstruction processes, the neural network is trained independently. The training platform comprises an Intel Xeon-E5-2640v4 CPU and an Nvidia RTX-2080ti GPU, and the training time is 2 h 10 min.

Figure S6 illustrates the loss functions during training (please see the “CBD” curves). The training loss converges well and the validation loss does not show overfitting, indicating a successful training. As for comparisons, we also show the performance of training when the AUTOMAP is not real-valued (see Figs. S6 and S7, and Supplementary Section for further information). We find that if an ONN fails to represent the complete real-value domain, it will perform poorly or even fail to reconstruct images. Therefore, the ability of amplitude modulation and coherent detection of the OCDC is necessary.

### In-situ BPC

The OCDC is analog computing hardware. The imperfections of the fabricated devices have a large impact on the final numerical accuracy. Even if we can calibrate the OCDC by measuring the modulation curve (transparency vs. applied voltage) of every modulator, the computing results still deviate from the desired ones when all modulators work simultaneously. We use the BPC method to further minimize the deviations of the hardware. In contrast to the previous ONN in-situ training methods such as^46^, our BPC is used to fine-tune parameters from a computer-pretrained network. Instead of updating the parameter as a whole, the BPC updates parameters independently and it is suitable for the OCDC to reach higher numerical accuracy. Assume *k* × *k* parameters are to be fine-tuned. The computing complexity of BPC is O(*k*^2^), which is at the same order of magnitude as the in-situ training method^46^. For a temporally multiplexed OCDC, the forward propagation is formulated as

4 $$y_i = \mathop {\sum}\limits_{j = 1}^M {x_{i,j} \cdot w_j,i = 1,2,...,N}$$

where *M* is the number of branches used for dot product. It is 3 in the experiment. The time step *N* is 250. By defining the mean square error (MSR) of the results as the loss function, we can calculate the derivatives of the loss function (*L*) on the hardware-represented weights (*w*).

5 $$\frac{{\partial L}}{{\partial w_j}} = \frac{2}{N}\mathop {\sum}\limits_{i = 1}^N {\left( {y_i - \hat y_i} \right)} \cdot x_{i,j}$$

where $\hat y$ is the result of the desired dot product. Update these weights by changing the applied voltages on the modulator, the MSR of the results is minimized (more examples of BPC are illustrated in Fig. S10). Since the BPC is conducted on a digital computer, we estimate the overhead of this process. By assuming a single-core CPU working at 4-GHz clock speed, the backpropagation theoretically takes only 0.18 μs to finish. Considering the accuracy improvement provided by the backpropagation, such overhead is acceptable. Note that the forward propagation and backpropagation of the OCDC are all linear. Nonlinear distortions cannot be eliminated by this method. Therefore, such nonlinearity imposes a limitation for BPC (further discussed in the Supplementary Section).

### Conducting the AUTOMAP by the OCDC

The linear part of the first FC layer and the first convolutional layer of the AUTOMAP is experimentally conducted by the OCDC chip. In the first FC layer, the size of the input vector is 1 × 32768 and the size of the weighting matrix is 32768 × 8100. They are decomposed to small parts with the size of 1 × 3 and 3 × 8100, respectively. The values from the input vector are loaded to the “slow mod.” modulators and the values from the matrix is loaded to the “fast mod.” modulators. Because of the massive weight matrix of the FC layer, it is impractical to conduct all operations with the OCDC working at a modulation rate of 100 Hz. The OCDC carries out operations for three typical parts in the input vector: the corner, the center, and the edge (see Fig. S11 for further information). For the convolutional layer, we conduct the convolution with the first kernel (5 × 5). The kernel is flattened to a vector and the input feature map is rearranged to a matrix with the size of 25 × 16384. Then, the decomposition method is similar to that of the FC layer. The OCDC conducts linear parts, and the nonlinear activation functions are implemented in the computer. From all the experimental results, we calculate the normalized standard deviation of residual errors introduced by the OCDC. It is averagely 0.0076 for the FC layer and 0.0104 for the convolutional layer. In the image reconstruction processes, we impose these experimental deviations to every layer of the AUTOMAP as additive noise to simulate the situation that the neural network is completely conducted by the OCDC. Corresponding results are provided in Fig. 5 and Figs. S12–14. In addition, the quality of image reconstruction with different levels of computing error is simulated and discussed in Supplementary Section.

## Supplementary information

Supplementary information for Optical coherent dot-product chip for sophisticated deep learning regression

## Data Availability

The brain images used for training and evaluation were obtained from the MGH-USC HCP database (https://db.humanconnectome.org/).

## Code availability

Source code is available from the corresponding author upon reasonable request.
