# Supplementary material for: Optical coherent dot-product chip for sophisticated deep learning regression
Source: Light Sci Appl. 2021 Nov 1;10:221. doi: 10.1038/s41377-021-00666-8 (PMC8560900; doi:10.1038/s41377-021-00666-8)
Supplement: Supplementary file 1 — Supplementary information for Optical coherent dot-product chip for sophisticated deep learning regression [file 41377_2021_666_MOESM1_ESM.docx]

**Supplementary information for Optical coherent dot-product chip for sophisticated deep learning regression**

Shaofu Xu^1^, Jing Wang^1^, Haowen Shu^2^, Zhike Zhang^3^, Sicheng Yi^1^, Bowen Bai^2^, Xingjun Wang^2^, Jianguo Liu^3^, Weiwen Zou^1^*

*^1^State Key Laboratory of Advanced Optical Communication Systems and Networks, Intelligent Microwave Lightwave Integration Innovation Center (imLic), Department of Electronic Engineering, Shanghai Jiao Tong University, 800 Dongchuan Road, Shanghai 200240, China*

*^2^State Key Laboratory of Advanced Optical Communications System and Networks, Department of Electronics, School of Electronics engineering and Computer Science, Peking University, Beijing 100871, China*

*^3^Institution of Semiconductors, Chinese Academy of Sciences, Beijing 100083, China*

**Correspondence to: wzou@sjtu.edu.cn.*

**S1. Chip fabrication and characterization**

The on-chip devices in this paper are designed and fabricated on the silicon on insulator (SOI) platform with 0.13 μm mask technology. Besides the thermo-optic version of OCDC used in the proof-of-concept experiment, an electro-optic (EO) version of OCDC, which is consistent with the architecture, has also been fabricated and measured for future demonstration in high-speed scenarios. As shown in Fig. S1a, the silicon Mach-Zehnder modulator here works under the carrier-depletion mode which ensures the 3 dB EO bandwidth of above 24 GHz, as indicated in Fig. S1b. Therefore, it is promising to improve the computing speed for about several gigahertz. The insertion loss of each modulator is ca. 5dB.

**Fig. S1** **a,** The top view of the EO version of the OCDC. A modulation branch is marked with a red block, including two cascaded electro-optic modulators and a tail phase shifter. **b,** Bandwidth measurement of fabricated EO modulators. The 3-dB bandwidth is over 24 GHz. **c, d,** the schematic illustration (**c**) and optical image (**d**) of the 1x9 cascaded directional coupler (DC) array.

The dot-product process consists of both multiplying operation within one modulation branch and adding operation of all branches, which may result in calculation deviations if uneven power splitting happens. A cascaded directional coupler (DC) array is introduced to achieve even power splitting. The schematic is shown in Fig. S1c. Ideally, each branch should obtain 1/9 of the input power, and that will make the power splitting ratio distribution 1/*i*, where *i* is the port number. Moreover, a residual power level of P_r_=~30% is allocated to the monitor port. In that way, the actual coupling strength of each DC can be described as P_r_/*i*. In our design, the width of the silicon bus waveguide of the cascaded DC structure is 450 nm and the gaps between bus waveguide and each branch are set to be 250 nm, with the coupling length varying from 5.2 μm to 8.1 μm. Figure 2d in the main text shows the test results of the normalized power at each output port. One can tell that the power deviation is less than 1.2 dB among all ports, showing a considerable result with uniformed power splitting.

**Fig. S2** Deployment of the monitors on the chip. **a,** Schematic of the OCDC, where the monitors are marked with green triangles. The optical power for monitor is divided from the output port of each modulator via a 1:9 MMI coupler. **b,** The top view of three modulation branches on the thermo-optic OCDC, showing six monitors on the chip. **c,** Design, fabrication and measurement results of the 1:9 MMI.

We deploy a coupler at the output of every modulator so that we can monitor and characterize the performance of all modulators individually. As shown in Fig. S2, optical power for monitoring is divided from the output of the modulator using a 1:9 asymmetric multimode interferometers (MMIs). Such splitting ratio is constructed by simply breaking the symmetry of the multimode region [1]. The proposed asymmetric MMI power splitter is shown in Fig. S2c. Compared to the conventional symmetric power splitter, the only difference is that the symmetry of the multimode region is broken by removing its bottom left corner (marked with a red dashed rectangle). Such a minor structural change causes a dramatic redistribution of the optical field thus leading to an uneven power splitting by changing the value of *L_r_*. We randomly chose four identical 1:9 MMI and test the power splitting ratio. The results are found to be very close to the design target (9.0), as indicated by the bottom right table in Fig. S2c.

Using these monitors, we measure the maximal optical power of every modulator by changing the bias voltage to their maximal transparency. Results are shown in the table of Fig. S3. The imbalance of the 9 branches in the thermal-optic OCDC is around 2.6 dB and it is around 3.8 dB for the electro-optic OCDC. The difference of optical power between the thermo-optic OCDC and the electro-optic OCDC comes from the deviation of fiber edge coupling at the light input port.

**Fig. S3** Tables of measured optical power after every modulator on the thermo-optic OCDC (**a**) and the electro-optic OCDC (**b**), respectively. It is found that the uniformity and insertion loss of the thermo-optic modulators is superior than the electro-optic ones.

The method of module packaging is illustrated in Fig. S4 and Fig. S5. For the OCDC, there are 36 signal pads and the space of the pads is 250 μm. In consideration of the ease of usage and highly configurable feature, the high-speed socket with up to 100 positions (Samtec, ERF8-RA) is selected as the optimal solution for the compact module, as shown in Fig. S4. The 60 DC pads have been connected using a flexible printed circuit (FPC) connector. In order to match the silicon-based waveguide mode field and increase the coupling efficiency, the tapered fiber with ~3.5 μm mode field is used for optical coupling. For RF transmission path configuration, a transmission line printed circuit board (PCB) with preferable mechanical strength is required to place high speed socket. And a transmission line AlN circuit board (ACB) is employed to connect the OCDC and the transmit line PCB. In order to ensure the high frequency performance, the impedance matching of the transmission line is required. The high frequency performance of the RF transmission path is measured as shown in Fig. S5. Insertion shows the measurement scheme in which the pinboard with GPPO connectors and the high frequency probe are used. It can be seen that the -3-dB bandwidth can all reach up to 7.8 GHz for the center line and the edge line, respectively. In this sense, the intrinsic performance of the OCDC is ensured.

**Fig. S4** The packaged OCDC with measurement setup. **a**, the photograph of packaged module in which the DC and RF measurement device is shown. **b** and **c** are the input and output coupling tapered fiber of the OCDC, respectively.

**Fig. S5** The RF transmission characteristics of the module. **a**, the RF transmission path configuration including a high-speed socket, a transmit line PCB and a transmission line ACB. **b**, The frequency response of the whole RF transmission path. For the edge line and the center line, the -3-dB bandwidth can reach up to 7.8 GHz which can support the performance of the OCDC. Insertion is the measurement scheme.

**S2. Performance of the AUTOMAP in incomplete real-value domains**

The numerical basis for most regression tasks is the complete real-value domain, as shown in Fig. 1. However, the capability of practical ONNs of realizing complete real-value domain may be limited by the physical constraints. For example, architectures using non-coherent light to represent values are not able to conduct operations with negative input values. With balanced detection, weights and outputs can be real-valued. Similarly, coherent architectures use optical amplitude to represent real-valued inputs and weights. However, using single-ended photodetection is unable to output negative values. Besides, architectures that rely completely on intensity modulation, intensity attenuation, and intensity detection can only conduct operations with non-negative inputs, weights, and outputs. The numerical domain of the above situations is not complete. Different from these architectures, the OCDC proposed in this work can represent real-valued inputs and weights with amplitude modulators. It can also output real values because the output amplitude is biased by optical interference prior to detection.

**Fig. S6** Loss functions of the AUTOMAP during training. **a-c**, Training losses of different architectures on different reconstruction processes. Note that the training loss implies fitting ability of a neural network. The fact that the CBD architecture outperform other architectures indicates the necessity of the complete real-value domain for deep regression tasks. **d-f**, Validation losses, which implies the generalizability of a neural network.

In this section, we investigate the performance of the AUTOMAP if the real-value domain is not complete. As discussed above, there are four situations: coherent architectures with single ended intensity detection (CID), non-coherent architectures with balanced detection (NCBD), architectures with intensity-only capability (InOn), and coherent architecture with biased detection (CBD, this work). The AUTOMAP is modified to simulate the incompleteness of the real-value domain in different situations except for the CBD situation. To simulate the performance of CID architectures, absolute value operation is imposed to the results of linear part of each layer. For NCBD architectures, the activation function of FC layers is replaced with ReLU to give non-negative input values. For InOn architectures, absolute value operation is imposed to the weights and output results of each layer. Every modified network is trained independently on three reconstruction processes (MF, vPDS, and Radon).

**Fig. S7** Reconstructed images by these four architectures (CBD, CID, NCBD, and InOn). **a-c**, The MF process, the vPDS process, and the Radon process, respectively. The ground truth image is offer on the top. On the right side of each reconstructed image, residual error image is attached (values are amplified by 2 times for visibility).

Results are shown in Fig. S6 and Fig. S7. From the loss functions of training and validation, we observe that the CBD architecture can obtain the best convergence among these four situations. The NCBD and CID architectures can also converge but with inferior performance. The InOn architecture is unable to converge. Such difference in performance is obviously shown in Fig. S7. If the real-value domain is incomplete, the quality of image reconstruction will be much inferior. The InOn architecture even fails to conduct image reconstructions.

**S3. Calibration of the OCDC**

The first step of calibration is to control the bias voltages of every modulator. The modulator is push-pull configured and the input signal is a saw-like wave. If the modulator is set to the null point, the output waveform should be a standard sine wave. The drift of bias voltage leads to harmonic distortion of even orders. We calibrate the bias voltages such that the second-order harmonic distortion of the output waveform reaches the minimal.

The second step is to obtain the constructive inference among different branches. The optical combiner used in the OCDC is cascaded directional couplers (DCs), which is symmetrically the same as the optical splitter. As shown in Fig. S1c, the combining ratio is 1:1, 1:2, …, 1:9, respectively. Optical interference is occurring at every DC, which can be formulated as

$A'_{n+1}=\left[\begin{matrix} \sqrt{\frac{n}{n+1}} & \sqrt{\frac{1}{n+1}} \end{matrix}\right]\left[\begin{matrix} A'_{n} \\ A_{n} \end{matrix}\right].$ (s1)

Eq. s1 describes the interference of the *n*-th DC. *A_n_* is the complex amplitude of *n*-th input port. The output field of interference *A^’^_n+1_* is the input of the next DC. *A^’^_1_*= *A_0_.* After 9 DCs, the final output amplitude is formulated as

$A_{out}=\frac{1}{\sqrt{10}}\sum_{n=0}^{9} A_{n}$ (s2)

It is found that the cascaded DCs perform equivalently as a single ten-port optical combiner with uniform combining ratios. Eq. s2 indicates that we can calibrate the phases of all branches by measuring the final output field instead of the output field of every DCs. With this conclusion, it is possible to observe the in-phase condition of all branches. With the modulators tuned to maximal transparency, the final output field reaches the maximum when all branches are in-phase.

**S4. Limitation of backpropagation control**

In general, the performance of the backpropagation control (BPC) method is limited by two factors: noise and nonlinear effects. The influence of random noise can be eliminated by increasing the time step (*N* in Eq. 4 and Eq. 5) used for BPC. Because the noise can cancel each other out at the averaging process. However, it is not recommended to use a very large time step. With increased *N*, The BPC will consume more time and more resources to calculate the gradients. Besides, although the BPC can be very precise, the numerical accuracy of forward propagation is still affected the system noise. The extra accuracy of BPC does not contribute to better performance of dot product.

The second factor of the performance limit is the nonlinearity introduced by the ‘fast mod.’ modulators. Since we assume that the OCDC performs dot product linearly, the BPC is only capable to compensate the linear deviations of the forward propagation. However, nonlinearity always exists, in practical modulators. Despite the inherent nonlinearity of phase shifter, in our experiment, the major part of nonlinearity comes from the imbalanced phase modulation, i.e. phase shifts of the upper arm and the lower arm are not equal. As stated in the Methods, we use an approximate *P_π_* for simplicity. Given that the actual *P_π_* for every thermal phase shifter is not the same, such approximation will introduce nonlinearity.

Suppose the accurate *P_π_* for upper arm and the lower arm are $P_{\pi}^{(u)}$ and $P_{\pi}^{(l)}$, respectively. The loaded power for both arms is $\pm P_{0}$. The output optical field of the push-pull modulator is formulated as

$\begin{matrix} E=\frac{1}{2}\left(e^{jP_{0}\pi\frac{1}{P_{\pi}^{u}}}+e^{jP_{0}\pi\frac{-1}{P_{\pi}^{l}}}\right) \\ =\cos\left(P_{0}\pi\frac{1}{P_{\pi}^{u}}\right)+j\sin\left(P_{0}\pi\frac{1}{P_{\pi}^{u}}\right)+\cos\left(P_{0}\pi\frac{1}{P_{\pi}^{l}}\right)+j\sin\left(P_{0}\pi\frac{-1}{P_{\pi}^{l}}\right) \\ =\cos\left(\frac{P_{0}\pi}{2}\left(\frac{1}{P_{\pi}^{u}}+\frac{1}{P_{\pi}^{l}}\right)\right)e^{j\frac{P_{0}\pi}{2}\left(\frac{1}{P_{\pi}^{u}}-\frac{1}{P_{\pi}^{l}}\right)} \\ \text{=}\cos\left(\frac{P_{0}\pi}{2}\Sigma\right)e^{j\frac{P_{0}\pi}{2}\Delta} \end{matrix}$ (s3)

We can find a residual phase shift besides the standard push-pull modulation (the cosine function). This residual phase shift term distorts the linearity when it is detected by a photodiode. Suppose the reference optical field for photodetection is $A_{ref}$. The output photocurrent is formulated as

$\begin{matrix} I_{pho}\propto\mathrm{Re}\left\{\left(\cos\left(\frac{P_{0}\pi}{2}\Sigma\right)e^{j\frac{P_{0}\pi}{2}\Delta}+A_{ref}\right)\cdot\left(\cos\left(\frac{P_{0}\pi}{2}\Sigma\right)e^{-j\frac{P_{0}\pi}{2}\Delta}+A_{ref}\right)\right\} \\ =A_{ref}^{2}+\cos^{2}\left(\frac{P_{0}\pi}{2}\Sigma\right)+2\cos\left(\frac{P_{0}\pi}{2}\Sigma\right)\cos\left(\frac{P_{0}\pi}{2}\Delta\right) \end{matrix}$ (s4)

which is not a perfect square expression. The square root of Eq. s4 is not linear to $cos\left( \frac{P_{0}\pi}{2}\Sigma\right)$. A method to minimize such nonlinearity is to lower the deviation of *P_π_* ­between upper and lower arms, i.e., the *Δ* in Eq. s4. This depends on further advancement of photonic integration technologies. Another solution is to measure the *P_π_* parameter for every phase shifter so that we can compensate for such deviations by changing the applied power, $P_{0}$. However, this introduces more challenges to the calibration process and the encoding process from pixel values to voltages. Again, we note that the forward propagation suffers from system noise. Over-precise BPC is not helpful for the overall performance of dot product computing. There should be a balanced point between resource consumption and performance.

**Fig. S8 a,** The schematic of the spatially multiplexed OCDC. The input light splitter is the same as the OCDC presented in this work. After the ‘fast mod.’ modulators, the optical signals are again split into multiple ‘slow mod.’ banks. A bank carries out a dot product operation, and a PD outputs the result. **b,** The structure of homodyne detection. Using this structure, the output photocurrent is proportional to the amplitude of input optical field, e.g., $I_{pho}\propto A_{ref}\cdot\sum_{i} A_{i}\cdot cos(\Delta\varphi)$.

**S5. Potential scalability of the OCDC**

The basic concept of the OCDC is to reuse an optical dot-product core to perform matrix multiplications and convolutions of arbitrary size. While the temporal multiplexing of the OCDC is demonstrated in the main text, the OCDC can be also spatially multiplexed. The spatial multiplexing architecture of the OCDC is illustrated in Fig. S8a. the basic idea is to split the optical signals from the ‘fast mod.’ modulators to multiple ‘slow mod.’ modulators. As a single ‘slow mod.’ bank can conduct an operation of dot product, spatially multiplexing the OCDC can perform multiple dot product operations at once. In the architecture, the most energy-consuming and technically challenging part is the fast modulators. Therefore, the spatially multiplexed OCDC only duplicates the ‘slow mod’ part and photodetection part, to reuse the signals from fast modulators. The advantage of parallelism of optics can thus be exploited. Theoretically, with larger number of duplicates, energy efficiency of the OCDC becomes higher [2].

A key point of spatial multiplexing of the OCDC is low-loss waveguide crossings since they are largely adopted in the architecture. With current planar crossing technologies, insertion loss lower than 0.1 dB/crossing and crosstalk lower than 35 dB are obtainable [3], guaranteeing the implementation of large-scale spatially multiplexed OCDC. For more aggressive goals, multi-planar waveguide crossing can be adopted [4, 5]. The insertion loss and the cross talk can be further reduced. In the spatially multiplexed OCDC, the BPC is still feasible. Compared with the coherent ONN architecture based on Mach-Zehnder interferometer (MZI) mesh, BPC of the OCDC is straightforward and simple. In the MZI-based ONN, the deviation of each output port is influenced by every phase shifter in the mech. The whole weight matrix should be upgraded at the same time to minimize the deviation [6, 7]. However, in the OCDC, every output port is independent. One can easily determine which modulator introduces the deviation and calibrate them independently.

Another factor regarding the scalability is the computing density. The basic computing unit of the OCDC is an MZM (or MZI). Compared with microrings (MRRs), MZMs are much larger on footprint. So, the capability of conducting real-valued operation is at the cost of the computing density. According to the evaluation by T. Ferreira de Lima et al, MRRs can achieve a compute density about 50 TMAC s^-1^ mm^-2^ whereas MZI-based ONN can only achieve 0.56 TMAC s^-1^ mm^-2^.

**S6. The performance of AUTOMAP under different error level**

Since the numerical accuracy of ONNs are highly relevant to the quality of deep learning regression. Here, we discuss how does the computing error influence the quality of image reconstruction. We generate different levels of random noises and add them into the ideal AUTOMAP to simulate that the AUTOMAP is carried out by the OCDC with different computing error. In this simulation, computing errors of the FC layers and the convolutional layer are the same. For every noise level, 200 images are reconstructed to show the stochastic result. The result is illustrated in Fig. S9.

**Fig. S9** The error of reconstructed images with different levels of computing error. **a,** The Misalign reconstruction process. **b,** The vPDS reconstruction process. **c,** The Radon inverse transform reconstruction process.

It is observed that with higher computing error, the integral error of image reconstruction is higher. Except for the low-error region, the error of image reconstruction approximately grows linearly with the computing error. It is inferred that the quality of image reconstruction should be promoted linearly by achieving better numerical accuracy. When the numerical accuracy is high enough, the reconstruction quality is most governed by the capability of the neural network model.

**Supplementary references**

1. Q. Deng, L. Liu, X. Li, and Z. Zhou, Arbitrary-ratio 1 × 2 power splitter based on asymmetric multimode interference, Optics Letters 39, 5590-5593 (2014).
2. M. A. Nahmias, T. Ferreira de Lima, A. N. Tait, H. Peng, B. J. Shastri, and P. R. Prucnal, Photonic multiply-accumulate operations for neural networks, IEEE Journal of Selected Topics in Quantum Electronics 26, 7701518 (2020).
3. Y. Ma, Y. Zhang, S. Yang, A. Novack, R. Ding, A. Eu-Jin Lim, G. Lo, T. Baehr-Jones, and M. Hochberg, Optics Express 21, 29374-29382 (2013).
4. J. Chiles, S. Buckley, N. Nader, S. Nam, R. P. Mirin, and J. M. Shainline, “Multi-planar amorphous silicon photonics with compact interplanar couplers, cross talk mitigation, and low crossing loss”, APL Photonics 2, 116101 (2017).
5. J. Chiles, S. M. Buckley, S. Nam, R. P. Mirin, and J. M. Shainline, “Design, fabrication, and metrology of 10 × 100 multi- planar integrated photonic routing manifolds for neural networks”, APL Photonics 3, 106101 (2018).
6. T. W. Hughes, M. Minkov, Y. Shi, and S. Fan, Training of photonic neural networks through in situ backpropagation and gradient measurement, Optica 5, 864–871 (2018).
7. M. Y. S. Fang, S. Manipatruni, C. Wierzynski, A, Khosrowshahi, and M. R. Deweese, Design of optical neural networks with component imprecisions, Optics Express 27, 14009-14029 (2019).
8. T. Ferreira de Lima, A. N. Tait, A. Mehrabian, M. A. Nahmias, C. Huang, H. Peng, B. A. Marquez, M. Miscuglio, T. El-Ghazawi, V. J. Sorger, B. J. Shastri* and P. R. Prucnal, Primer on silicon neuromorphic photonic processors: architecture and compiler, Nanophotonics 9, 4055-4073 (2020).

**Fig. S10** Residual error during BPC control. **a-c,** The adopted weights are [1, 1, 1]. Standard deviation of the residual error and corresponding gradients of the weights are shown in the figure. **d-f,** The adopted weights are [0.2, 1, 0.8].

**Fig. S11** The input data preparation of the first FC layer. In the first FC of the AUTOMAP, the input images are flattened to a vector. For MF and vPDS processes, the input data includes a real image and an imaginary image. For Radon process, the input data is a real-valued sinogram. Sizes of these images are marked in the figure. Since the size of input vectors (32768) is significantly larger than the size of OCDC, it is extremely challenging to implement the complete matrix vector multiplication (32768×8100) experimentally with the modulation rate of 100 Hz. We used three typical parts (corner, center, and edge) on the image for OCDC calculation as a proof of concept. **a,** The data preparation for the MF process. After flattening, the pixels on the corner, the center and the edge of the real image are used for experimental implementation. The blocked values are the pixel values from these parts. **b,** The data preparation for vPDS is the same to that shown in **a**. **c,** The Radon process only has a real image. So, the locations of these three parts are different from those in **a** and **b**.

**Fig. S12** More examples of reconstructed images on MF process. Values of the error is amplified by 10 times.

**Fig. S13** More examples of reconstructed images on vPDS process. Values of the error is amplified by 10 times.

**Fig. S14** More examples of reconstructed images on Radon process. Values of the error is amplified by 10 times.
